# The health-disease process and the family health strategy: the user's perspective^1^

**DOI:** 10.1590/0104-1169.0002.2496

**Published:** 2014

**Authors:** Débora de Souza Santos, Elainey de Albuquerque Tenório, Mércia Zeviane Brêda, Silvana Martins Mishima

**Affiliations:** 2 PhD, Adjunct Professor, Universidade Federal de Alagoas, Maceió, AL, Brazil; 3 RN, Prefeitura Municipal de Salvador, Salvador, BA, Brazil; 4 PhD, Professor, Universidade Federal de Alagoas, Maceió, AL, Brazil; 5 PhD, Full Professor, Escola de Enfermagem de Ribeirão Preto, Universidade de São Paulo, WHO Collaborating Centre for Nursing Research Development, Ribeirão Preto, SP, Brazil

**Keywords:** Family Health Strategy, Health Promotion, Community Health Nursing, Nursing

## Abstract

**OBJECTIVE:**

to analyze the meanings Primary Health Care users attribute to their health-disease process and the services used.

**METHODS:**

this qualitative research uses the focus group technique to interview two groups of users the service monitors. The first is a group of elderly people and the second of pregnant women. To analyze the meanings, the discourse analysis technique and the reference framework of health promotion are used.

**RESULTS:**

the group of elderly, being mostly female arterial hypertension and diabetes mellitus patients, visualizes the health-disease process as the evolution of human existence controlled by divine power, signifying the health service as a blessing in the control of the disease. The Group of young pregnant women signified health as the ability for self-care and disease as the disability for that purposes, considering the Primary Health Care service as responsible for the recovery of individual and family health.

**FINAL CONSIDERATIONS:**

the users demonstrated dissatisfaction with bureaucratic and vertical relations present at the health services. In each group, it was observed that the meanings for health and disease and meanings of the health service the users elaborated can be related.

## Introduction

In the last two decades, the Unified Health System (SUS) has advanced in different aspects, including the expansion of the Family Health Strategy (FHS), the reconfiguration of the management and funding of the system and the legal institutionalization of the deliberation spheres for popular participation. Some authors^(^ 1 ^-^ 2 ^)^, however, signal weaknesses that make it difficult to consolidate a care model based on health promotion.

A model focused on health promotion presupposes that the health-disease process results from social, economic, cultural, ethnic/racial, psychological and behavioral determinants, which can contribute to the emergence of diseases and constitute risk factors for the population and configure their quality of life indices^(^ 3 ^)^. Thus, health promotion requires articulation among the different social sectors besides health, which guarantee conditions for the users' empowerment, towards social control in the management of knowledge, techniques, power, physical, financial and human resources, focused on acting on the determinants of health and disease^(^ 4 ^)^. Hence, we consider that the health needs the users bring and translate contribute to the constitution of health practices, related to the configuration of the care model^(^ 1 ^)^.

As part of the discussion about care models, the health needs or health problems the users bring demand health actions and services with a view to their solution. The combination of these health practices configures a health care model. The health needs the users present are constituted based on their interpretation of health and disease. This interpretation receives influence from countless biological, social, psychological and economic determinants and conditioning factors, in a constant constitution process of subjectivities^(^ 5 ^)^. It has been observed, however, that the health professionals still ignore these aspects, which receive strong influence from the biomedical model, where the work process is impersonal, centered on the cure of an established disease, on the subject's hospitalization and fragmentation, withdrawing their autonomy regarding their health-disease process, which is that necessary to strengthen health promotion^(^ 6 ^)^.

When considering the interpretations and meanings formulated by the users, the health promotion services combine three fundamental characteristics: ethical, to the extent that ethical relations between user and service are only developed when the users are seen as individuals and social agents of their own changes; pragmatic, as the users only age through dialogue and participate based on the experience of daily practices; and critical reflection about the ideas and conceptions that guide the health practices among the different actors^(^ 7 ^-^ 8 ^)^. Based on these premises, questions emerge about the meanings the users attribute to their health-disease process and to the Family Health Strategy service in view of the reality in Maceió-AL, Brazil?

In 2010, despite the improvement in comparison with the year 2000, the socioeconomic indicators of the state capital of Alagoas indicated a population in which 40.67% lived on less than a quarter of a minimum wage. The rates of illiteracy and unemployment figured around 11.32% and 12%, respectively. At the same time, the child mortality rate corresponded to 18.6%, higher than the Brazilian average, while the mortality rate due to transmissible diseases increased from 59.1% in 2009 to 63.1% in 2010. As opposed to this reality, in 2010, only 29.53% of the population in Maceió-AL was covered by the Family Health Strategy^(^ 9 ^)^.

In that sense, the objective in this study is to analyze the meanings the users attribute to their health-disease process and the Family Health Strategy service, as formulated by the users in their sociocultural context. This study contributes directly to the understanding of how the users signify health, disease and health services. These meanings are related to the daily reality of the health services, valuing integrality and popular participation as re-structuring guidelines of the care model aimed at health promotion in this particular reality.

## Methods

Qualitative study, involving users from a Family Health Unit in the city of Maceió, Alagoas, Brazil, between January and May 2011, through focus groups. This study received approval from the Research Ethics Committee at Universidade Federal de Alagoas (process 013414/2010-20).

The focus group is a research technique that consists in holding a collective interview with a homogeneous group, planned for the purpose of interaction among the subjects in the discussion of a central theme. Thus, through the statements, the study permitted capturing the meanings the FHS users attributed to health, disease and the health service^(^ 10 ^)^.

For that purpose, the focus groups were planned with users who participated in two distinct health education groups at the Family Health Service as, according to the professionals working at the service, their bond with the service is stronger. These were the elderly group and the pregnant women group. Invitations were elaborated for the users who participated in these groups, informing about the date and time when the group was held and the theme to be discussed, which were delivered with the help of the community health agents. On the day, place and time set, we started the interview with the groups, using the following triggering questions: "What does it mean to experience health?", aiming to capture the senses and meanings attributed to health and disease and "What do you think of the FHS health service", aiming the capture the senses and meanings related to the health service.

According to the methodological criteria of the focus group, the groups were held by a coordinator and an observer, so as to guarantee the participation of all subjects, using a semistructured script.

The collective interviews were recorded, transcribed and analyzed based on the Discourse Analysis^(^ 11 ^)^ method, adopting the reference framework of Health Promotion as the theoretical model for analysis^(^ 12 ^)^. The discourse is the "the activity that produces meanings and takes place in the interaction among speakers"^(^ 13 ^)^. The meaning is social and historically constructed and takes form in language. The discourse analysis aims to obtain the meanings of the texts, relating them with the religious, philosophical, sociopolitical, legal and economic conjuncture that permeates them. Names of planets were used for the participants from group 1 and names of flowers for the participants from group 2, with a view to preserving their identity.

## Results

One meeting was held with each group. The meeting with the first group took 41 minutes and 18 seconds, while the meeting with the second group took 30 minutes and 54 seconds.

The first focus group held involved five users, mostly women, predominantly elderly, between 55 and 75 years of age, who attended the group of arterial hypertension and diabetes mellitus patients at the Family Health Service. The second group involved seven young women, between 21 and 29 years of age, who participated in the group of pregnant women at the same service.

**Figure 1:**
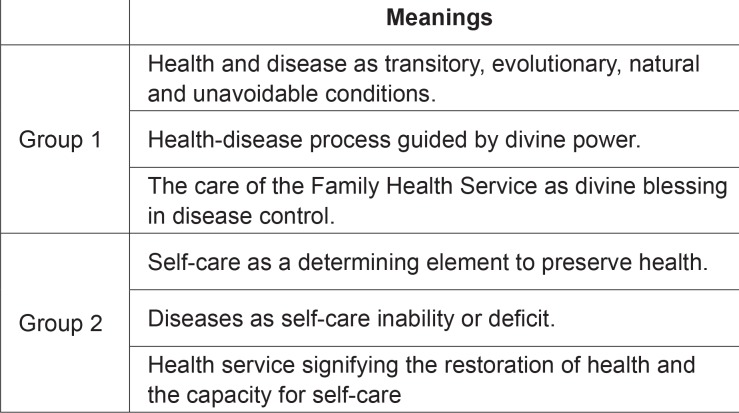
Synthesis of the meanings emerged in the focus groups. Maceió, AL, Brazil, 2011

The discourse analysis followed the logic of the production of meaning^(^ 10 ^)^: first, words and expressions the participants used were identified, based on which the figures and themes were apprehended in thematic phrases. For the thematic phrases to make sense, however, as recommended in Discourse Analysis, the thematic phrases were related with the group they emerged in, in the time and space they were produced in, with the participating users and their power relations reproduced in the discourse. Figure 1 summarizes the meanings apprehended in each focus group:

## Discussion

### Analysis of focus group1: Health is like when we are young

The elderly users, represented as group1 in Figure 1, do not put forward health and disease from a dualistic viewpoint, opposing the phenomena, but signify health and disease as transitory, evolutionary, natural and unavoidable conditions. They characterize health as physical completeness that allows the individual to resist adversities, permeated by the feeling of individual pleasure and more characteristic of young people. Disease is characterized as a disabling phenomenon, which causes suffering and dependence, more characteristic of the elderly phase of life they experience, as observed in the statements: *Health is like when we are young, right? You go where you want, everything is good, everything is healthy, but when we're old, we go somewhere today and tomorrow we wake up beaten, tired (Uranus, 65 years). You have to die one day, I'm not gonna stay to gain root* (Earth, 72 years).

It is understandable that, as elderly people with chronic illnesses, various physical limitations affect them, influencing their self-perception as people who were healthy once and are ill today and experiencing the imminence of death^(^ 13 ^)^. This form of signifying health and disease is based on the determination of the existence of live organisms, who are programmed to be born, live and die^(^ 14 ^)^. Death is not an exclusive fact of old age though, but it is certain that "if we survive until old age we will die, not because we are old but because we are human"^(^ 14 ^)^.

This interpretation of the group approximates the idea that, in the course of their life, the individuals are susceptible to the different internal and environmental risks that unbalance the organism, requiring effects to re-establish a new equilibrium. The modifications provoked to restore the bodily balance over time gradually change it, and the old, weaker body results from these processes. Consequently, it is admitted that disease is part of health which, within this perspective is but "the ability to get ill and recover"^(^ 15 ^)^.

One user explains that this *health-disease process is guided by divine power: Health is a blessing of the heavenly father... Because, you see, there are people who cannot live, the diet is weak, I myself... I thank Jesus Christ for the health he gives me!* (Saturn, 55 years).

According to that user, only the divine power justifies survival amidst the adversity of the social and economic conditions, as a form of salvation, in line with the reference framework of health promotion, which considers that adverse conditions are unable to produce health. In the sacred conception of health and disease, gaining health is a synonym of salvation^(^ 14 ^)^. In that sense, the Family Health Service gains the meaning of *divine blessing in disease control and in the promotion of a healthy life: The service where I arrived to get treatment was that one and I value it, it's a blessing for me, since I'm there no one has ever been bad to me... On another occasion I got there dying, but I was treated like a queen* (Neptune, 75 years).

This view of the health service as God's gift reflects in the users' passive posture towards the service as, thus, the services loses its characteristic of a conquered and legally guaranteed right for the user. The relation of submission that is established can also be observed when the users mention "asking the consent" of the health agent or the reception staff to be attended by the health service.

The meaning of health as a divine blessing grants the health professionals great power over the users, contributing to the configuration of a vertical relation between health professional and community, the first of whom hold the knowledge, while the users are ignorant people who are solely responsible for feeling bad^(^ 16 ^)^. In the magic-religious conception of health, disease is considered as a divine punishment for having broken the divine laws, leaving the individuals with a passive posture of submission before the divine will to cure or eliminate the evil. Thus, for the users, in view of their need, there is no alternative but to accept the peregrination in search of care at the service as part of the remission process of guilt and the achievement of cure^(^ 16 ^)^.

The observation of the users' statements reveals this phenomenon, when they report that they do not mind the lack of specialized services, lack of medication or long waiting times. Despite being entitled to priority as elderly, they resignedly wait for their turn in the line, taking the blame for not being attended because they did not wait quietly, without complaining, or for not arriving in time to get a place in the line: *... Because I do not get there, to go first... A lot of confusion has already happened in there, you know? Because of that... There's a line there, you have to wait in the line!* (Mars, 70 years). *The elderly people, they get there and are attended first... I wasn't attended now because I get there beyond the time, right? The doctor is attending, but I arrived late, so I leave. Because I didn't arrive in time* (Uranus, 65 years).

The organization of the daily agenda and scheduling of appointments are the responsibility of the health service workers. The users should participate in their construction, reducing the bureaucracy and vertical relations between users and services^(^ 17 ^)^. The statements that the users take the blame for not adjusting to the queue, however, reveal how the service can exert its power over the subjects, discouraging their ability to participate and their autonomy. In that sense, the encounter between community and service gains a controlling mechanism, which reproduces ideologies and social products^(^ 17 ^)^, different from the FHS's recommendations for the constitution of a care model focused on health promotion, with a care logic centered on the relation between user and service, based on mutual respect, valuation of subjectivities and the subjects' autonomy^(^ 18 ^)^.

On the menu of the FHS's program actions on the daily agenda, the elderly group members report that they benefit from the distribution of medicines, forwarding for surgeries, pressure measurement, capillary glucose test, medical and nursing consultations, vaccines and participation in educational activities. The users value the health agents' care quite highly, as they feel accompanied, even when they are at home: *It's good for me. What's good is the care... To look at the diabetes, the body looks at our finger, weighs us, no matter how many times I tell him to he weighs me, measures my pressure, forwards me to the doctor... When I don't go to the doctor I get it from the nurse ...*(Uranus, 65 years). *She has already forwarded me for one operation... I was operated on... Everything started there... The girls who work there like me a lot. But thanks God...* (Mars, 70 years). *After I went to that service there, if I go for a consult, I am attended, because my health agent goes there to our house. Then I get very satisfied, because at those other services we are confronted with a queue... *(Neptune, 75 years).

In these statements, the users also highlight how they value respectful, considerate and problem solving treatment during care. Based on these criteria, they establish relations with the services that are marked by positive or negative feelings. In that sense, it is common for the users, without technical criteria to assess the professionals' performance, to use daily parameters to assess the workers, such as lovability, good will and complacence^(^ 18 ^)^. Based on these criteria, they can develop positive or negative relations, depending on the successful solution of their health needs, which will determine the continuation and compliance with the services offered^(^ 19 ^)^.

In the users' discourse, the development of positive feelings can be observed in the terms "being treated like a queen", "the staff likes me", "the elderly are attended first". On the other hand, the development of negative feelings was observed in the statements of other users who reported feeling rejected by the services, when they lacked information, at times of conflicts with the team, in the scheduling of appointments that did not fit into their routine and regarding the long waiting time: *When I showed the boy the document, like... I felt rejected and left, right? There, I didn't go back, right?* (Earth, 72 years). *I myself, right? There's one thing to complain about there... the vision screening... so many days to schedule it and she scheduled it on the 10th, the 10th is a Sunday! *(Saturn, 55 years).

When the relation between user and service limits any of the parties and the feelings do not get due space in the daily service routine, the health actions tend to be strict, vertical and excluding, that is, they dehumanize the service. Even positive feelings, if not properly discussed and addressed between the users and workers, can produce a relation based on compassion, informality, dependence on personal judgments and values, highlighting the asymmetric nature of the relations^(^ 19 ^)^, which goes against the principles of humanization in health, autonomy and the co-accountability of the actors who construct the health services. Therefore, the feelings produced in the relation between user and service, as witnessed in the statements, reveal a great transformative potential, if addressed in the sense that "the person whom we share the trajectory with is considered as an authentic interlocutor and not simply as an "object" of our initiatives"^(^ 20 ^)^.

Hence, this picture indicates the need to permit and enhance democratic spaces for decision making, which value the meanings of the subjects involved in the health production process, with a view to the establishment of solidary bonds that permit the reversal of the care model in the context of the FHS^(^ 20 ^)^.

### Analysis of focus group 2: Experiencing health means doing well for yourself and other people

The group of women indicated *self-care as a determinant element in the preservation of health, *through healthy practices of hygiene, food, sleep and physical exercise, avoiding stress and drugs use, as observed in the statements: *...It means living well, eating well, taking care of one's own health (Rose, 22 years). It means not drinking because it's not good for your health, not smoking... Yes, I think that's it (Violet, 23 years). If your house is not hygienic... your health goes down the drain* (Dahlia, 26 years).

Self-care is defined as one of the aspects of healthy life. The users put it forward as the individual ability to perform actions for oneself or in the environment, which keeps the body functioning appropriately to perform one's daily functions and be well. The self-care perspective presupposes that health is the way through which people manifest their process-based existence, coexisting with other beings, communicating with the world, exercising the "human desire to know, to seek the truth and do well for oneself and others"^(^ 21 ^)^.

For the FHS, self-care is a necessary prerogative to achieve health promotion, as it reinforces the individual's autonomy, turning him/her into a responsible agent in the search for quality of life. The self-care discourse, however, should be analyzed in the light of the neoliberal society, which it is part of, so as not to drop into individualism, blaming the individuals for their disease and exempting the State from its responsibility to provide the resources needed for the individuals to promote health. In other words, self-care is important in a health promotion model but should be considered as part of a broad and democratic policy, which promotes the quality of life in view of the social determinants and conditioning factors of health^(^ 22 ^)^.

Consequently, in view of the social determinants and conditioning factors in the territory the users live in, marked by precarious housing, basic sanitation, education and leisure activities, besides high unemployment, under-employment and violence rates, it is inferred that the users consider good hygiene and food practices for themselves and their families as fundamental aspects for their individual and family health.

In addition, it is relevant to weigh that the women are increasingly providing their families with material needs. The female double work journey is widely disseminated and known, also in the poorer population groups, overburdening the social role of women, like the interviewees^(^ 23 ^)^. The literature also indicates that women are the group that most visit health services for themselves and the family, particularly Primary Health Care^(^ 24 ^)^. In that sense, the signification of health as the ability for self-care may be related to the female need to be apt for work and for the organization of their homes, in view of providing the family core with material and affective support.

Thus, preserving their own health appears as an important factor to take care of family members and people close to them, generating feelings of pleasure, satisfaction, happiness, joy and courage: *Happiness, knowing that he's well, not at risk... The most important is to be healthy... Because if I'm not healthy how am I going to take care of him? *(Points towards her son on her lap) (Dahlia, 26 years). *It means living well, being happy... Experiencing health is doing well for oneself... and for other people... *(Chrysanthemum, 21 years). *Experiencing health is... Being courageous and being like that without any fuss. It means playing, running, smiling, talking, it's the desire to do things and that's it* (Daisy, 29 years).

In this case, self-care and health provide positive mutual feedback, so that self-care produces health and health grants individuals the pleasure of autonomy to take care of themselves and the people close to them, the mastery of their own life. Self-care appears not only as important to preserve health, but as a measure of health.

They describe the set of self-care actions as a 'healthy' way of life - not using drugs, healthy eating, exercising, going to the doctor, being hygienic, among others. Nevertheless, the healthy lifestyle discourse as a self-care model rests on an underlying individualistic health promotion practice, despite its positive relation with life, as it contains a fixed set of rules, prescribed by the expert, who ignores the users' life context and subjectivity^(^ 25 ^)^. The user perceives this separation in this testimony: *I am always taking care of myself and treating my illnesses... Ah, eating well... although I don't eat very well, right* *(laughs) *(Azalea, 24 years).

While health as the ability for self-care and care of close people produces feelings of pleasure and happiness in the users, *disease means the self-care inability or deficit,* provoking sadness, death, stress, inability, suffering and anticipated mourning: *You sit in the corner sad (ill), downcast, you just stay there living other people's happiness, waiting for the day when your time comes* (Daisy, 29 years). *Ah it's very bad (being ill)... because you know that you're going to die, that you've got little time left to live...* (Violet, 23 years).

In view of the inability for self-care during a disease, the loss of mastery over one's own health, the suffering and weakness it provokes, the users delegate the mastery over the care of their body to the health service and hope to recover their self-care ability. Thus, *the meaning of the care the FHS delivers is the recovery of health. Because how are we going to live without health? You'll always be going to the doctors, hoping that one day you'll get ill* (Violet, 23 years). *To me, experiencing health means... attending the doctor... going to the doctor* (Magnolia, 28 years).

They visit the service when they are ill, that is, when they feel weak and unable to perform self-care. On that occasion, they dismiss their responsibility and expect to recover their health at the service, in this case the FHS. Nevertheless, they feel dissatisfied with the care the service delivers, because it does not attend to their expectations. Some users report that some employees treat them in a hostile manner, that they do not consider the weaknesses and difficulties they face to get access to the service (disease, hunger, absence from work, queues), prioritizing specific population groups, such as the elderly, hypertensive patients, diabetics, children and pregnant women.

Care is based on daily appointments and rests on a strict and fixed timetable, which even excludes the possibility of care delivery to priority population groups depending on the day of the week. This is the classical public care model, asymmetric and vertical, where humanization becomes practically impossible, as it emphasizes the techno-bureaucratic logic, far from the establishment of relationships, of encounters, that is, it hampers the establishment of dialogical spaces. The configuration of care in the FHS does not advance in breaking with this situation, as its design rests on a set of guidelines, attributions and productivity protocols that restrict the workers' self-management power, contributing to the strictness in the service supply^(^ 19 ^)^. This situation entails a negative critical assessment by the users: *Bad is that most people are not attended well when they arrive, they come from their home without eating, are absent from work... There just are not enough people here... But not these ignoramuses!* (Daisy, 29 years). *Because my sister, she's tall and needs a dentist and she can't come to the dentist, because only pregnant women, elderly or children are allowed* (Azalea, 24 years). *A service near my house is no use, cause it doesn't do the things... we need, in case of a disease (Daisy*, 29 years*).*

According to the users, however, the community health agents and educative actions are positive counterpoints of the FHS service. The community health agents are valued because of the bond they establish with the users, through the home visits, helping to schedule appointments and examinations, that is, permitting access. The educative actions, on the other hand, are valued because of their informative and transformative nature, helping the users with self-care and family care: *Because there are health agents who come to our home, what we asked the doctor, she takes the exam, it may take time, sometimes it takes time, but sometimes it doesn't, I think that's normal. I like it* (Violet, 23 years). *It would be good if it was offered (education group) after we had the baby too, right?* (Magnolia, 28 years). *Because afterwards, when we have the baby, they don't even call us anymore, they invite the pregnant women more and after we have it we don't want to learn anymore, right?* (Dahlia, 26 years).

Thus, the group of women manifested critical ability to assess the adequacy and problem-solving ability of the services, indicating weaknesses and strengths of the FHS to produce affective bonds, promote self-care and recover health.

## Final considerations

This study permitted the identification of different meanings for the health-disease process and the health services offered by a FHS in Maceió, in two distinct groups of users, who live within the same territory. It was observed in both groups that, inside each group, the way the users signify health and disease may be related to the meaning they produce for the health service. The question is raised to what extent the health service determines the way the users signify their form of thinking health and disease and their health needs. Despite different macro-political initiatives in the SUS towards a model based on health promotion, the users' discourse reveals that the biomedical model remains hegemonic in the micro-political organization of the service, revealing the need for further studies that permit understanding and intervening in this space to revert this model. In addition, existing dialogical spaces need to be strengthened and the creation of new spaces needs to be encouraged, where FHS users and workers can express their perceptions, feelings and desires regarding the health practices, considering the subjective experience of their health-disease process in the health actions, revealing the users' autonomy for self-care in health promotion. The health professionals' valuation of the users' subjectivities, including health education actions and articulation with intersectoral support to the population contributes to revert the care model in this reality by enhancing the production of health practices that are committed to health promotion, integrality and participation.
